# Structural Disorder Provides Increased Adaptability for Vesicle Trafficking Pathways

**DOI:** 10.1371/journal.pcbi.1003144

**Published:** 2013-07-18

**Authors:** Natalia Pietrosemoli, Rita Pancsa, Peter Tompa

**Affiliations:** 1 National Centre for Biotechnology (CNB-CSIC), Madrid, Spain; 2 Department of Bioengineering, Rice University, Houston, Texas, United States of America; 3 VIB Department of Structural Biology, Vrije Universiteit Brussel, Brussels, Belgium; 4 Institute of Enzymology, Research Centre for Natural Sciences, Budapest, Hungary; Peking University, China

## Abstract

Vesicle trafficking systems play essential roles in the communication between the organelles of eukaryotic cells and also between cells and their environment. Endocytosis and the late secretory route are mediated by clathrin-coated vesicles, while the COat Protein I and II (COPI and COPII) routes stand for the bidirectional traffic between the ER and the Golgi apparatus. Despite similar fundamental organizations, the molecular machinery, functions, and evolutionary characteristics of the three systems are very different. In this work, we compiled the basic functional protein groups of the three main routes for human and yeast and analyzed them from the structural disorder perspective. We found similar overall disorder content in yeast and human proteins, confirming the well-conserved nature of these systems. Most functional groups contain highly disordered proteins, supporting the general importance of structural disorder in these routes, although some of them seem to heavily rely on disorder, while others do not. Interestingly, the clathrin system is significantly more disordered (∼23%) than the other two, COPI (∼9%) and COPII (∼8%). We show that this structural phenomenon enhances the inherent plasticity and increased evolutionary adaptability of the clathrin system, which distinguishes it from the other two routes. Since multi-functionality (moonlighting) is indicative of both plasticity and adaptability, we studied its prevalence in vesicle trafficking proteins and correlated it with structural disorder. Clathrin adaptors have the highest capability for moonlighting while also comprising the most highly disordered members. The ability to acquire tissue specific functions was also used to approach adaptability: clathrin route genes have the most tissue specific exons encoding for protein segments enriched in structural disorder and interaction sites. Overall, our results confirm the general importance of structural disorder in vesicle trafficking and suggest major roles for this structural property in shaping the differences of evolutionary adaptability in the three routes.

## Author Summary

Vesicle trafficking systems are fundamental among cellular transport mechanisms; various cargo molecules are transported via different coated vesicles to their specific destinations in every eukaryotic cell. Clathrin-coated vesicles mediate endocytosis and the late secretory route, while the COat Protein I and II (COPI and COPII) vesicle trafficking routes are responsible for the bidirectional traffic between the ER and the Golgi apparatus. Despite similar basic principles, regulatory mechanisms and structural features of the three systems, their molecular machinery, functions, and evolutionary characteristics vastly differ. We investigated and compared these three routes and their basic functional protein groups from the structural disorder point of view, since disordered protein regions could provide a broad variety of functional and evolutionary advantages for them. We found that structurally disordered protein segments are most abundant in the clathrin system, which might explain the observed inherent plasticity, increased adaptability and exceptional robustness of this route. We support our hypothesis by two analyses on protein multi-functionality and tissue specificity, both being indicative of evolutionary adaptability. Clathrin pathway proteins stand out in both measures, with their disordered regions being largely responsible for their outstanding capabilities.

## Introduction

The well-organized network of vesicle trafficking pathways is the basic communication mechanism between the different intracellular compartments and the environment, and as such it is crucial for the efficient transport of macromolecules within and between cells and their environment. Transport vesicles deliver various cargo molecules (proteins, lipids, signalling molecules, etc.) to the plasma membrane and to specific membranous compartments, while being also responsible for maintaining the appropriate protein and lipid composition of various organelles. There are several specialized routes present in all eukaryotic cells playing distinct roles, being responsible for different directions of traffic between various source and target membranes of several organelles and for carrying various types of cargo. These systems have their own well-conserved macromolecular machinery with a specific coat protein complex on the surface of their vesicles, which usually lends the name to the whole system.

While endocytosis and the late secretory route are mostly mediated by clathrin-coated vesicles, the early secretory pathway from the ER (endoplasmatic reticulum) to the Golgi apparatus and the retrograde transport backwards are mediated by the COPII (COat Protein II) and COPI (COat Protein I) routes, respectively [1]–[4]. Although the protein machinery of these pathways vastly differs, with almost no common members, they do share several main structural and mechanistic characteristics.

In all three cases there is a specific multisubunit protein coat complex on the outer surface of the vesicles which can self-assemble as a lattice collecting and concentrating the appropriate adaptor-cargo complexes into membrane patches. This process is not only responsible for cargo selection, but through the generation of membrane curvature, it also enhances the budding and fission of vesicles [5]. The coat also determines the shape and size of vesicles, which vary a lot in the three main systems [6], [7]. The architecture and assembly mechanism of these cages have been extensively studied by electron microscopy and crystallography [7]–[11], and are extensively reviewed [5], [6], [12], [13]. Despite limited sequence similarity, proteins involved in coat assembly have a common underlying structural design using β-propeller domains and long stretches of α-solenoid motifs as basic building blocks [5]. The clathrin cage assembles from triskelion assembly units, trimers of clathrin heavy chains (CHCs), that are centered on cage vertices with their long α-solenoid legs extending along whole edges until reaching adjacent vertices, while also overlapping with the legs of neighboring triskelions [8], [9]. The N-terminal β-propeller domains of CHCs project inwards in order to interact with the adaptor proteins residing on the surface of the vesicle membrane [14], [15]. Clathrin light chains (CLCs) form an extended single helix on the surface formed by the interhelical loops of the CHC α-solenoid legs with their termini occupying ambiguous localizations [8]. Despite containing the same types of domains as CHCs, the COPII cage architecture is much different from that of clathrin. In this case, the assembly unit is a heterotetramer of two Sec13-31 (Protein transport protein Sec13 and 31) dimers forming a long rod by the α-solenoid legs of those, enclosed by two β-propeller domains at each end. The cage is cuboctahedron shaped with the rods forming the edges and four rods converging into each vertex without any overlaps between them [7], [16], [17]. The COPI cage shows a design intermediate between COPII and clathrin, with its domain organization and vertex interactions similar to those of COPII, and the triskelion shape (with curved legs converging from three-fold centers) resembling clathrin coat subunits [10].

In all three pathways vesicles are uncoated after formation, i.e. their cage-forming scaffold proteins and the adaptor proteins are stripped off either stepwise (COPII and clathrin) or at once (COPI). The precise timing of this procedure is still under debate [18]. The pathways also share the dependency of the coat assembly on different small GTPases of the ARF/SAR (ADP-ribosylation factor/Secretion-associated RAS-related protein) family, as well as on their corresponding activating or nucleotide exchanging factors [19]. Cargo handling also shows many features in common. Adaptor proteins link the scaffold to the cargo and to the membrane, and communicate with other accessory proteins involved in other functions necessary for the formation and fission of the transport vesicle (e.g. deforming and sensing membrane curvature, causing scission, and up- or down-regulating any of the previous steps) [20], [21]. In addition, cargo-specific receptor proteins are also present in all three systems.

Other similarities between these routes include the basic mechanisms of vesicle transport (driven by motor proteins along the actin cytoskeleton elements) and vesicle fusion with the target membrane. The key players of vesicle fusion are SNARE (Soluble N-ethylmaleimide-sensitive factor attachment protein receptor/SNAP receptor) proteins in all the three systems. SNAREs behave like molecular engines; they generate the pulling force required for placing the two membranes close enough for fusion. Instead of using ATP in force generation, a huge conformational change occurs when the SNARE protein in the vesicle membrane interacts with the appropriate SNARE proteins in the target membrane (also providing specificity for the fusion process). The four SNARE coiled-coil homology domains assemble into a stable four-helix bundle, which is then disassembled in an ATP-dependent manner [22], [23]. The basic regulatory mechanisms of fusion are also common between the different systems. For instance, there are multisubunit tethering complexes or huge coiled-coil tethers [24] that help COPI and COPII vesicles to get close enough to the target membrane for the SNARE proteins to interact and arrange fusion [18], [25].

Despite this array of mechanistic, structural, and regulatory similarities, there are fundamental functional and evolutionary differences between the clathrin-mediated system and the other two. While COPI and COPII are essential for the viability of eukaryotic cells (without any of them the transport between the ER and the Golgi is completely abolished) and they only occur in the ER-Golgi-ER routes, the clathrin system seems to be less indispensable and more broadly relied on. Knock-outs of certain main clathrin components, such as AP-2 (Adapter-related protein complex 2) are unviable in case of multicellular organisms [26]. On the other hand, yeast cells could get along without AP-2 [27] or even without clathrin [28], but not without some other clathrin pathway associated adaptors, such as the epsin-homologs [29], HIP1 (Huntingtin-interacting protein 1) and Hip1R (Huntingtin-interacting protein 1-related protein) [30]. Trypanosomes for instance, do not have any AP-2 like proteins, yet they rely on endocytosis [31]. Instead of implying that clathrin-mediated routes are not crucial, these observations emphasize their vast plasticity providing alternative ways for the cargo to get to the right place, by slightly altering the original pathway. In general, the clathrin-mediated system shows more plasticity and robustness than COPI and COPII, which from an evolutionary point could translate into more adaptability.

There are several differences in the complexity of the three pathways too. In the COPI and COPII systems, the adaptor subunits are part of the multisubunit coat complex and there is only one set of them for each pathway [32]. However, there are slightly different versions or isoforms for some of the subunits in both systems showing different cargo specificity or differential localization, which suggests distinct functional roles for the variously composed coat complexes [33], [34]. Instead, the clathrin system adaptors comprise a highly diverse and dynamic set of proteins, which may share similar functions (e.g. binding the clathrin coat and the cargo at the same time), or play individual roles in the assembly and transport of vesicles [21], [35]. They can participate in different routes, showing preference for different sorting signals and organelle membranes, sorting different cargo types into the same population of vesicles cooperatively, or recruiting cargo into different populations of vesicles on the same membrane in a mutually exclusive manner. Clathrin adaptors form extensive interaction networks on the membrane donor surface, many of them having copious known interaction partners.

Although the functional and evolutionary differences between the systems have been previously studied, the inherent structural characteristics that could account for them have not been fully explored. Some previous works allude that long regions of certain clathrin-associated adaptor proteins are disordered, unstructured or unfolded [36]–[39], but this phenomenon has never been investigated in a systematic manner. In a large-scale study protein transport was suggested to have the strongest correlation with structural disorder [40]. In our view structural disorder could have a pronounced role in certain functionalities important for vesicle trafficking systems, which would mean that their proteins could be at least partly accounted for the observed correlation. Interestingly, in the same study transport mechanisms in general were found to be depleted in predicted disordered regions. This would be logical because transportation of ions and small molecules requires a huge variety of large multi-pass membrane proteins (ion channels and different transporters) and these are usually very well-structured and consequently predicted as completely ordered. We believe that the presence of such disordered/unstructured regions may be a key phenomenon in vesicle trafficking; these disordered protein segments could account for many of the functional and evolutionary differences of the transport routes discovered so far, and hence, their abundance, as well as their locations and functions, should be adequately studied.

Intrinsically disordered proteins and regions lack a well-defined structure; they function via an ensemble of possible conformations [41], [42]. Being freed from the restrains of maintaining a folded structure, disordered protein segments show increased tolerance against mutations [43], which confers them the possibility of fast evolutionary changes. Indeed, structural disorder could allow for many functional and structural advantages for vesicle trafficking proteins. Due to their enlarged capture radius, disordered regions could offer the ability to bridge large distances via the “fly-casting mechanism” of protein binding [44] promoting effective assembly of the vesicle coat. In this mechanism loosely structured protein segments reach out and bind their partners from larger distances due to their many exposed protein interaction motifs. Since short linear protein interaction motifs [45], [46], posttranslational modification sites [47], [48], and tissue-specific disordered binding regions of splice variants [49], [50], usually reside in disordered protein segments, these regions could be especially important in facilitating specific binding to partner proteins and in displaying important regulatory roles [21], [38]. All the above mentioned characteristics of disordered regions, along with other advantages they provide – such as their conformational freedom and their ability to bind many interactions partners (moonlighting [51]) – make them excellent candidates for the efficient assembly [52] and transport of macroscopic organelles.

Given all the possible implications and advantages intrinsic disorder can have on the different vesicle trafficking mechanisms, studying its role in them is of indisputable importance. To our knowledge, the abundance of disordered regions was only improperly assessed for proteins in the clathrin pathway by secondary structure prediction methods [38], inappropriate for identifying structurally disordered protein segments. Furthermore, the presence of structural disorder was not addressed in the other two major vesicle trafficking systems. Hence a quantitative assessment of protein disorder content using adequate methods is still lacking. In the recent years, the field of intrinsic structural disorder has flourished and the importance of unstructured protein regions has been finally recognized in many key cellular processes [41], [42]. In this work, we present a systematic study of protein disorder in all three main vesicle trafficking systems using adequate methods. The quantification of intrinsic structural disorder together with a comparison of disorder content in the different pathways should aid in understanding how these structural characteristics affect the functional and evolutionary features of vesicle trafficking proteins.

## Methods

### Collection of proteins of the major vesicle trafficking systems in human and yeast

Clathrin route proteins were collected from comprehensive reviews [20], [35], while the main components of the COPI and COPII vesicle coats were collected from their specific literature [3], [4], [53]. Proteins involved in vesicle fusion regulation (multisubunit tethering complexes, coiled coil tethering proteins, SM (Sec1/Munc18-like) proteins and other regulatory proteins) were compiled from fusion-specific literature [18], [24], [54], just like SNARE proteins [22]. In case of the two latter groups, proteins and their corresponding functions were collected regardless of their specific pathway, because they function in a very similar manner in all cases [18]. Following literature-based data collection, we extended our protein datasets with their interaction partners reported to function in the three systems by the Universal Protein Knowledgebase (UniProtKB) [55]. We also included proteins annotated with vesicle trafficking-related terms of the Gene Ontology (GO) Database [56] (namely GO:0048208, GO:0012507, GO:0006892, GO:0030126, GO:0030130, GO:0030132, GO:0030136 GO:0048205, GO:0006890) after manually checking their UniProtKB annotation and the literature to make sure that they mainly function in vesicle trafficking routes. The resulting dataset contains only manually curated proteins.

### Collection of human and yeast complete proteome sequences

The protein sequences of the complete human (Homo sapiens) and yeast (Saccharomyces cerevisiae; strain ATCC 204508/S288c) proteomes were obtained from the UniProtKB release 2012_09) [55] and filtered for fragmented proteins and 95% sequence identity. Finally, 20213 human proteins and 6221 yeast proteins were used to calculate whole-proteome reference data.

### Protein classification schema

Whenever possible, the classification of proteins found in the literature was used. Seven large functional groups were defined: four of them were budding and fission-associated: i) coat proteins; ii) adaptors and sorting proteins; iii) enzymes and enzymatic activity related proteins; and iv) a general group for unclassified proteins. Only the proteins of these were classified according to the three main systems (Clathrin-mediated, COPI and COPII mediated). The other three functional groups correspond to fusion-associated proteins: v) SNAREs; vi) multisubunit tethering complexes; and vii) other fusion regulators. In case of the human proteins, an extra functional group of fusion regulators playing a specific role in the regulation of neurotransmitter transport was added. The unclassified group includes all the proteins for a given system that could not be classified into the first three budding and fission-associated groups but still take part in these processes, many of them being transmembrane cargo-specific receptors. GEFs (Guanine nucleotide exchange factors) were not included in the analysis since they were only reported to act on their specific small GTPase. Although sometimes highly specific for the process, these proteins were not considered as part of the transport vesicles themselves.

### Prediction of intrinsic disorder and disordered binding regions

Prediction of intrinsic protein disorder was carried out using the IUPred method [57], [58]. IUPred predicts intrinsic structural disorder using sequence information alone, based on the possible pairwise interaction energies (calculated from statistic potentials) between a given residue and the residues of the surrounding sequence windows. If possible favourable interactions prevail with the given protein segment, the amino acid is predicted to be ordered, otherwise it is predicted to be intrinsically disordered. The predictor provides a disorder probability value in the 0.0–1.0 range for each residue as an output. Using the standard 0.5 value as threshold, we mapped the prediction into a binary (“ordered” vs. “disordered”) classification at the residue level.

Prediction of disordered binding regions (DBRs) possibly involved in protein-protein interactions was carried out using the ANCHOR method [59], [60]. This method predicts putative binding regions within a protein sequence if they are intrinsically disordered in isolation and may undergo a disorder-to-order transition upon binding. As well as IUPred, the method is based on energetic estimations. The format of the output of this method is similar to that of IUPred and hence it was transformed in the same manner.

### Calculation of different measures to describe the disorder content of proteins

Standard measures generally used in the literature to describe the disorder content of proteins were calculated: i) relative disorder content or ratio of disordered residues (predicted number of disordered residues/total number of residues), ii) ratio of residues in Long Disordered Regions (LDRs) (ratio of residues belonging to continuous stretches of at least *k* disordered residues; *k* = 30, 50 and 100), and iii) ratio of residues in ANCHOR-predicted Disordered Binding Regions (DBRs). In case of the transmembrane proteins, residues belonging to the reported transmembrane segments were not taken into account for the calculation of any disorder metric.

### Statistical evaluation of differences in disorder measures

Comparisons of disorder metrics among the different groups of vesicle trafficking proteins (functional groups or complete systems) were performed using Wilcoxon Rank Sum Test. Comparisons of disorder measures in specific groups to the corresponding reference proteome values, were performed using Fisher's Exact Test.

### Identification of transmembrane segments and protein domains

Transmembrane regions were assigned according to UniProtKB [55] annotations, while protein domains and their corresponding locations were assigned using the PfamScan method [61] for all sequences. Default domain coordinates were assigned from the HMMER3 alignment coordinates using only high quality Pfam-A HMM profiles for the search.

### Identification of orthologous pairs of proteins

Orthology identification between human and yeast proteins was performed by the Inparanoid v7 program [62].

### Identification of non-vesicle trafficking interaction partners for all human budding and fission-associated proteins

Interaction partners with the highest confidence (confidence level = 0.9) were obtained from the STRING database 9.0 [63] for all budding and fission-associated human proteins. The resulting Ensemble identifiers were mapped to UniProtKB accession numbers and the corresponding sequences were obtained from the UniProtKB [55]. Two filtering criteria were applied to the data. In the first filtering step, interaction partners showing more than 70% sequence identity (according to the CD-HIT algorithm [64]) with any of the vesicle trafficking proteins listed in our database were excluded. Additionally, for each cluster of partners of the same protein with at least 70% sequence identity, only the one retained by CD-HIT (generally the longest one) was kept. In the second filtering step, a manual curation was performed on the resulting set of potential non-vesicle trafficking related interaction partners. Proteins involved in intracellular protein transport, endocytosis or vesicle trafficking according to their UniProtKB annotation were also removed. Proteins of the GTP-ase family (present in the three main routes) were excluded from further statistical analysis because of their non-specific nature and their extremely high number of identified interaction partners.

### Definition of the structural properties of tissue-specific exons within vesicle trafficking proteins

All tissue specific exons (TSEs) for the budding- and fission-associated human proteins of our dataset were collected from Wang et al. [65]. Exons were classified as being tissue specific using Wang et al.'s criteria: if they scored at least 0.25 on a 0–1 exon switch score scale (calculated based on the difference between the two most extreme tissue inclusion level values measured for the given exon [65]). Only budding- and fission-associated proteins were included in this analysis because they were the only ones that could be grouped into the three main pathways according to the adopted classification schema. 45 tissue specific exons were identified. These TSEs were mapped onto Ensembl transcripts (one transcript for each). One TSE was excluded because it was a non-coding exon according to Ensembl [66] (exon: chr1:54127505-54127724:-, corresponding to the YIPF1 gene). Structural disorder and disordered binding sites were predicted for the whole proteins by IUPred and ANCHOR, respectively, and the ratios of disordered residues within the regions corresponding to the TSEs and the predicted binding sites overlapping with them were calculated and identified.

### PDB search for complexes involving disordered protein segments

A comprehensive search in the Protein Data Bank (PDB) [67] was performed to identify complexes of distinct pairs of vesicle trafficking-related proteins in which the binding region of at least one of the partners is predicted to be intrinsically disordered by IUPred.

All data processing in this study was performed using scripts written in the Perl programming language. All analyses were implemented in the statistical analysis programming language R (www.r-project.org). The Pymol molecular graphics tool [http://www.pymol.org/] and the DOG2 protein domain representation tool [68] were also used for figure preparation.

## Results

### A comprehensive database of proteins involved in vesicle trafficking pathways

A comprehensive dataset of proteins functioning in the main vesicle trafficking systems in human and yeast was assembled. To the best of our knowledge, this is the largest and most complete such collection, containing 244 human and 162 yeast proteins.

### Intrinsic disorder in human and yeast vesicle trafficking proteins

Three different disorder metrics were used to explore the abundance of structural disorder and functionally important disordered sites in vesicle trafficking proteins: 1) the ratio of predicted disordered residues (hereafter referred to as disorder content), 2) the ratio of residues in predicted long consecutive disordered regions (LDRs) of different lengths (*k* = 30, 50 and 100 residues), and 3) the ratio of residues in predicted disordered binding regions (DBRs).

All collected and calculated data are provided in the Supporting Information (Table S1 and S2 for human and yeast, respectively). Each protein was identified by name and UniProt accession number, and classified according to the functional classification scheme (see Methods). The different types of disorder-related measures described in Methods as well as the number of transmembrane segments and different Pfam entities are provided for all proteins.

The number of proteins and the means and medians of all calculated disorder metrics for each functional class and each main pathway of vesicle trafficking proteins in human and yeast are summarized in Table 1. The ratios of proteins having LDRs of various length (k = 30, 50, 100) in the different functional classes and pathways, together with corresponding background data calculated on human and yeast complete proteomes are presented in Table 2.

**Table 1:** Calculated disorder measures of the different functional groups and the three vesicle trafficking systems for human (H) and yeast (Y).

	Number of proteins		Disordered residues (%)		Residues in DBRs (%)		Residues in LDRs (%) (k = 30 residues)	
			mean/median		mean/median		mean/median	
Functional groups	H	Y	H	Y	H	Y	H	Y
COAT	10	7	22.76/9.20	19.50/7.58	15.35/6.06	11.01/4.00	18.06/4.67	14.51/5.08
ASP	64	38	25.17/21.49	25.20/15.80	14.80/8.93	13.85/8.22	17.28/8.13	18.16/8.54
EARP	18	9	24.88/22.84	20.59/10.68	15.49/14.95	10.77/4.02	16.64/14.37	14.30/0
UCP	32	32	16.77/6.95	14.11/5.53	9.35/0.96	5.83/0	7.29/0	7.86/0
MSTC	44	42	6.01/4.75	8.96/5.08	2.39/0.74	3.58/0.74	1.94/0	4.12/0
OFRP	19	16	17.82/12.86	10.74/7.88	7.89/5.93	3.67/1.65	8.12/0	4.44/0
SNARE	37	24	23.74/18.26	26.97/24.63	9.31/6.91	13.58/11.51	5.72/0	8.93/0
NTSR	25	-	31.74/19.43	-	14.79/8.44	-	20.75/8.65	-
**Pathways**								
Clatrhin	71	38	27.98/23.33	27.84/22.58	17.19/11.58	15.58/11.50	19.40/13.35	20.30/10.41
COPI	22	16	13.33/6.90	13.92/8.22	6.11/0.83	6.28/0.95	6.80/0	8.13/0
COPII	31	32	17.52/6.78	14.07/6.25	10.45/2.88	2.88/6.08	9.42/0	8.45/0

The functional groups are COAT (coat associated proteins), ASP (adaptors and sorting proteins), EARP (enzymatic activity related proteins), UCP (unclassified proteins), MSTC (multisubunit tethering complexes), OFRP (other fusion regulatory proteins), SNARE (SNARE proteins) and NTSR (neurotransmitter transport specific regulators). H stands for human, while Y indicates yeast data in the given column.

**Table 2:** Fraction of proteins with disordered regions of various lengths for the different functional groups and pathways for human and yeast.

	Number of proteins		% Proteins with LDR (≥30 residues)		% Proteins with LDR (≥50 residues)		% Proteins with LDR (≥100 residues)	
Functional groups	Human	Yeast	Human	Yeast	Human	Yeast	Human	Yeast
COAT	10	7	60.00	57.14	50.00	57.14	30.00	28.57
ASP	64	38	60.94	57.89	45.31	44.74	37.50	28.95
EARP	17	8	70.59	50.00	58.82	50.00	52.94	50.00
UCP	28	27	28.57	22.22	21.43	22.22	14.29	11.11
MSTC	44	42	27.27	23.81	2.27	16.67	2.27	11.90
OFRP	19	16	36.84	37.50	26.32	18.75	26.32	0.00
SNARE	37	24	24.32	33.33	5.41	12.50	0.00	4.17
NTSR	25	-	60.00	-	40.00	-	12.00	-
**Pathways**								
Clatrhin	71	38	47	23	36	19	30	13
COPI	22	16	8	5	4	5	2	2
COPII	31	32	8	5	4	5	2	2
**Proteome**								
	20213	6621	45.60	35.45	33.11	24.51	18.18	12.49

Same abbreviations used as in case of Table 1. Last row corresponds to the whole proteome reference data (background).

Overall, proteins involved in vesicle trafficking tend to be slightly more disordered in human than in yeast proteins (mean disorder content of 20.85% vs 17.77%, respectively; one tailed Wilcoxon Rank Sum Test, p-value = 2.67E-02). The mean number of DBR residues is also higher for human than for yeast proteins (p-value = 1.80E-02). The overall disorder content of vesicle trafficking proteins (20.85% and 17.77% for human and yeast, respectively) is not statistically significantly different from that of the corresponding complete proteome (22.81% and 16.96% for human and yeast, respectively; Fisher's Exact Test) in either species.

### Intrinsic disorder in protein functional groups

**Figure 1:**
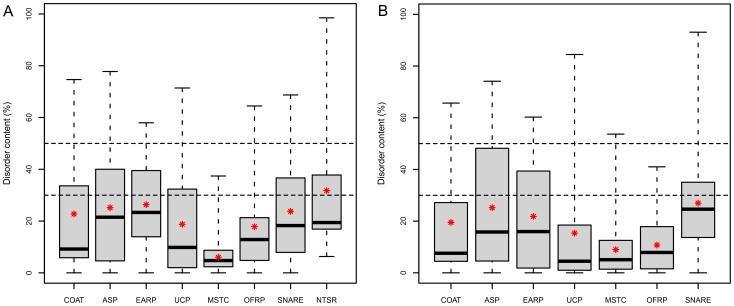
Disorder content for functional groups of proteins involved in vesicle trafficking. Disorder content (%) predicted by the IUPred method for the main functional groups of vesicle trafficking proteins in human (A) and yeast (B). Data are shown in Table 1. The main functional groups are COAT (coat proteins), ASP (adaptors and sorting proteins), EARP (enzymatic activity related proteins), UCP (unclassified proteins), MSTC (multisubunit tethering complexes), OFRP (other fusion regulatory proteins), SNARE (SNARE proteins) and NTSR (neurotransmitter transport specific regulators). The bottom and top border of the boxes represent 25% and 75% of the data respectively, while the bold line in the middle stands for the median (50%). The whiskers stand for the minimum and maximum values in the dataset. The mean is depicted by a red star. Proteins with disorder content (30% ≤ d.c. < 50%) are considered fairly disordered, while proteins with more than 50% disorder content are highly disordered.

The disorder content of the equivalent functional categories in human and yeast shows no statistical differences for any of the pairs as assessed by the Wilcoxon Rank Sum Test. All disorder metrics showed similar tendencies (and absolute values) for the main functional groups of the two species (Figure 1, Table 1). Thus, for the sake of clarity in the following section only data related to human proteins are described in detail.

Fusion regulation proteins are among the least disordered ones (Table 1) since their members are mostly subunits of large complexes. Proteins in the “coat” and in the “unclassified” groups (the latter containing many transmembrane cargo-specific adaptors) are also rather structured. These two groups, however, show larger deviations than the previous two. Although coat proteins form a completely folded, rigid, cage-like structure on the surface of the vesicles – and in good agreement with this, most of their subunits are predicted as completely structured using IUPred –, some of their subunits are predicted to be largely disordered. Such is the case of clathrin light chains (CLCs) (60.08% and 74.67% for clathrin light chains A and B, respectively), which in their bound form adopt a long α helical structure on the surface of the clathrin heavy chain (CHC) α-solenoid legs. However, in their unbound form they are likely to be highly disordered, which enables them to gain such an extended arrangement in the complex. Although to a lesser degree, the Sec31 subunit of the COPII type coat is also considerably disordered (33.61% and 27.40% for Sec31 A and B paralogs). The predicted disordered region matches well the long, low-complexity, proline-rich region of Sec31 proteins, which was shown to be unstructured even within the assembly unit by limited proteolysis [7] and also to mediate the interaction with the Sec23/24 adaptor subunit complex [69].

**Figure 2:**
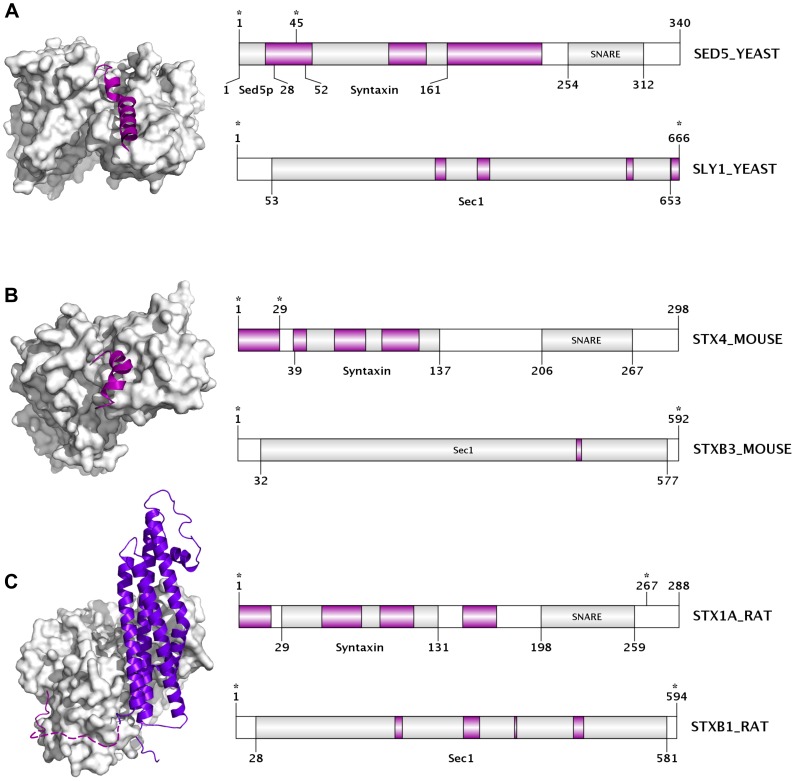
Interactions between the disordered N-terminal tails of SNARE proteins and their globular SM protein partners. Three PDB structures are presented showing the interaction between distinct pairs of the syntaxin-family SNARE proteins and their regulatory SM-proteins, an interaction that has been shown to positively regulate the SNARE complex assembly. The N-terminal of the SNARE partner is predicted to be mostly disordered (more than 50% of its residues) in the unbound form in all cases. (A) Interaction between the yeast syntaxin-family SNARE Sed5 N-terminal region, and SM protein Sly1 (PDB: 1MQS). (B) Interaction between the N-terminal tail of syntaxin-4 and syntaxin-binding protein 3 from mouse (PDB: 2PJX). (C) Interaction between syntaxin-1A (structure lacking the C-terminal transmembrane region) and syntaxin-binding protein 1 from rat (PDB: 3C98). Each interaction pair is represented by a PDB structure (left) and a domain map of the entire protein chain for both partners (right). The upper domain map corresponds to the SNARE protein, while the bottom one to the SM partner. In the structures, the disordered SNARE N-terminal tails are represented with cartoon style (magenta) while the partner molecule is in surface representation (white). In panel C, the remaining part of syntaxin-1A, which is not part of the disordered N-terminal tail, is coloured purple-blue, and those disordered residues of the N-terminal missing from the X-ray structure (10–26) are represented by a dashed-line. Names and lengths are provided for each protein in the corresponding domain map. Names and locations of their known Pfam domains (predicted by the PfamScan method) are also indicated. Regions predicted to be disordered (length of at least 3 consecutive residues) by IUPred are coloured in magenta, while the ordered segments are white (if not predicted to be part of a Pfam domain) or light-gray (if they are). Regions present in the PDB structures are marked by stars.

The “SNARE” group is composed of proteins containing at least one v- or t-SNARE coiled coil homology domain and various types of family-specific domains. Even so, they show a surprisingly high deviation in disorder content with a few of them being mostly disordered and others being well structured. The members of the syntaxin family of SNAREs have disordered N-terminal regulatory regions that can be used by their direct regulatory partners (e.g. SM proteins) to modify their function. Several PDB complexes demonstrate how the disordered syntaxin N-tail folds up when bound to a globular SM partner (Figure 2). Our results agree with the general view in the literature that SNARE motifs are unfolded in monomeric state, forming the four helix bundle only upon vesicle fusion [22], [23].

Although its median disorder content is only the second largest (21.49%), the group of “adaptor and sorting proteins” (ASPs) has the most highly disordered (>50%) members. This group is very diverse, containing completely structured subunits of larger adaptor complexes (such as the sigma and mu subunits of the AP complexes, the zeta subunit of the COPI coatomer complex, and the Sec23 subunit of the COPII coat adaptor) and also highly disordered adaptor proteins such as the epsins, DAB1 and DAB2 (Disabled homolog 1 and 2), HRS (Hepatocyte growth factor-regulated tyrosine kinase substrate) and NUMB (Protein numb homolog). The latter ones all have long LDRs enriched in binding motifs.

Interestingly, the group of enzymatic activity related proteins (EARPs) is the most disordered (22.84%). This might be counterintuitive at the first glance, since enzymes are thought to be typically well-folded proteins. Indeed, this is the case for domains carrying enzymatic activity or for small single-domain enzymes, like the small GTPases. However, their direct regulators, the long GAPs (GTPase activating proteins), for instance, are considerably disordered. The increased structural disorder content of GAPs in general was already reported and accounted to their long flexible inter-domain linker regions [40]. Other members of this group such as the synaptojanins, the AAK1 (AP2-associated protein kinase 1) and GAK (Cyclin-G-associated kinase) kinases, and auxillin, all contain very long disordered regions (at least one LDR≥100 residues) outside their structured domains and show more than 30% overall disorder content. In addition to their enzymatic activity, they are likely involved in protein-protein interactions or other disorder mediated functions as reflected by their enrichment in predicted DBRs.

Finally, the group of “neurotransmitter transport specific regulators” (NTSRs) contains distinct protein families: synaptotagmins, complexins, several neurotransmission-specific SM proteins, synaptophysin and tomosyn. Complexins are the most disordered family of our entire protein dataset (d.c. 76.25–98.51%), while the other members are highly ordered.

The statistical comparison of human functional groups against their whole proteome reference value (Table 2) showed that the group of ASPs is significantly enriched in proteins with LDRs of different length (LDR≥30, 50 and 100 amino acids; Fisher's Exact Test p-values: 9.89E-03, 2.84E-02, and 2.18E-04, respectively). Similar enrichment was found for the group of EARPs (LDR≥50 and 100 amino acids, p-values: 4.16E-02, and 2.15E-03, respectively). This enrichment is also valid for yeast proteins: ASPs are enriched in LDRs of all three lengths (Fisher's Exact Test p-values: 2.69E-03, 5.25E-03 and 5.64E-03, respectively) and EARPs are also enriched in LDRs≥100 residues (p-value = 1.84E-02) with respect to the whole proteome.

### Intrinsic disorder in the different vesicle-trafficking routes

**Figure 3:**
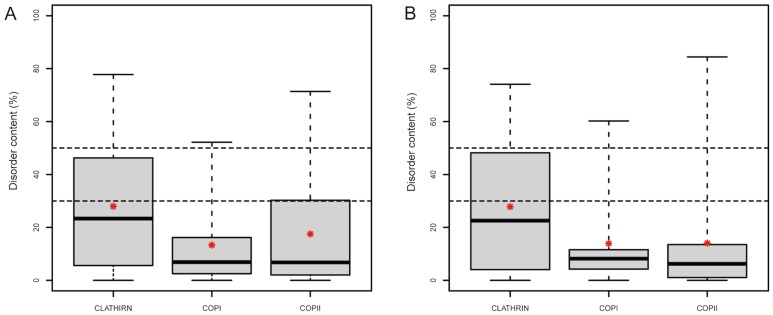
Comparison of systems in budding-associated functional groups of proteins. Comparison of disorder contents (%) predicted by the IUPred method between proteins involved in the three main vesicle trafficking systems for human (A) and yeast (B). Only data on budding and fission related proteins are presented here, since those could be reliably grouped according to the three main systems. Corresponding data are shown in Table 1. The bottom and top border of the boxes represent 25% and 75% of the data respectively, while the bold line in the middle stands for the median (50%). The whiskers stand for the minimum and the maximum of the data, while the mean is depicted by a small red star.

The disorder content of all budding- and fusion-associated proteins involved in the three main vesicle trafficking systems regardless of their functional classification was also compared (Figure 3, Table 1). Proteins associated with the clathrin-mediated route are the most disordered, with 23.33% and 22.58% median disorder content in human and yeast, respectively. This disorder content is significantly higher than the disorder content of COPI (9.20% and 8.22% in human and yeast, respectively; Wilcoxon Rank Sum Test; p-value = 9.89E-03 in human, 4.68E-02 in yeast) and COPII proteins (9.27% and 6.99% in human and yeast, respectively (p-value = 4.09E-02 in human, and 5.99E-03 in yeast)) in both species.

The disorder content of the proteins in the COPI and COPII routes is not significantly different in either species. Similarly, the average disorder content of the proteins in each of the three main pathways does not significantly differ between human and yeast (as assessed by the Wilcoxon Rank Sum Test).

The higher disorder content found in the clathrin-mediated route is partly due to the highly disordered clathrin light chains and mainly stemming from the several highly disordered proteins in the ASP and EARP groups. Overall, many of its proteins contribute to the observed effect. In case of the COPI and COPII pathways, most of the disorder contribution comes from a few outliers; namely, the huge, largely disordered Sec16 homologs for the COPII route, and a few highly disordered ArfGAPs (ADP-ribosylation factor GTPase-activating proteins) for the COPI route.

Only the clathrin-mediated route showed significant enrichment in proteins with LDRs of all three lengths (LDR≥30, LDR≥50 and LDR≥100) when compared to the corresponding whole proteome reference values for both species (Fisher's Exact Test; p-values: 3.85E-04, 1.66E-03 and 2.32E-06, respectively, for human and p-values: 9.40E-04, 6.28E-04 and 4.86E-04, respectively, for yeast).

### Measures for estimating the functional and evolutionary advantages of structural disorder in the clathrin route

In order to show that structural disorder provides enhanced evolutionary adaptability and plasticity in the clathrin pathway, two appropriate measures were introduced. The first measure reflects the capability of proteins to participate in multiple pathways and to mediate interactions with diverse partners (moonlighting). Although the correlation between moonlighting ability and structural disorder of proteins has been previously described [51], this work evidences that such relationship is present in proteins involved in vesicle trafficking and it compares the three main pathways from this point of view. The second measure introduced reflects the ability of proteins to show tissue specific functions/interactions. The increased preference of tissue specific exons (TSEs) for encoding disordered regions frequently embedding linear motifs, disordered binding sites or posttranslational modification sites has been recently demonstrated [49]. In order to investigate the proteins in the three main vesicle trafficking routes from this perspective, all their reported TSEs were collected and analyzed from both the structural and the functional point of views.

### Non-vesicle trafficking (off-pathway) interaction partners of proteins in the three main routes

High confidence, non-vesicle trafficking related (off-pathway) interaction partners were collected for all budding- and fission-associated human proteins in the dataset (Table S3). Out of the 363 collected interactions, most of them (297) belonged to the clathrin route, while 50 belonged to the COPII route and 19 to the COPI route. The mean and median of the identified interactions was also higher for the clathrin route (4.30/2.0) than for the COPII (1.72/1.0), and the COPI (0.95/0) routes. The number of such interactions for the clathrin proteins is significantly higher than that of COPII proteins (Wilcoxon Rank Sum Test, p-value = 0.016) and COPI proteins (Wilcoxon Rank Sum Test, p-value = 0.0063), while COPI and COPII proteins are not significantly different from this point.

For the most “interactive” proteins these values were compared with different measures of predicted structural disorder and disordered binding sites (Table 3). Interestingly, there is only one protein for the COPII (SEC13) and one for the COPI (COPB2) route out of the total 21, which have at least 5 off-pathway interaction partners, both of them functioning as coat complex subunits and showing very limited structural disorder content (6.52% and 9.19%, respectively). Out of the remaining 19 clathrin route associated proteins, there are 12 adaptor and sorting proteins, 4 enzymatic activity related proteins (the three dynamins and synaptojanin-1; 22.32–33.76% disorder content, all containing at least one LDR>100 residues), 2 coat components (clathrin light chain A with 60.08% disorder content and heavy chain 1 with 1.07% disorder content) both having 10 such interaction partners, and the unclassified Endophilin-A1 also showing 10 such interactions (disorder content ∼33%). Interestingly, among the 12 clathrin adaptors, the three AP-2 subunits show the least number of off-pathway interaction partners (mu (5), beta (6) and alpha-1 (7)) and also the lowest disorder content (4.16–14.74%). Among these three, only the most “interactive” alpha-1 has LDRs (>30) and predicted DBRs, and this protein has the highest disorder content also. The remaining 9 clathrin adaptors are altogether responsible for 135 (more than 1/3 of the total) off-pathway interactions, which is more than half of those mediated by the most “interactive” 21 proteins (266 interactions). These 9 clathrin adaptors do not form complexes, but act as single, they have quite high disorder content (15.65–74.16%), with most of them showing more than one LDR and plenty of DBRs (with the exception of the two beta-arrestins) and the majority of them being highly disordered (>50%).

**Table 3:** Data on proteins with at least 5 off-pathway interactions from the three main routes.

Gene Name	UniProt ID	Route	Num. of off-pathway interactions	Length	Disorder content (%)	Num. of LDRs (≥30)	Num. of DBRs	% of residues in DBRs	Functional Class
DAB1	O75553	CLTR	25	588	51.36	3	10	35.54	ASP
ARRB2	P32121	CLTR	25	409	15.65	0	1	2.44	ASP
DNM1	Q05193	CLTR	23	864	22.34	1	8	16.09	EARP
SEC13	P55735	COPII	22	322	6.52	0	0	0.00	COAT
ARRB1	P49407	CLTR	18	418	21.53	1	4	11.72	ASP
DNM2	P50570	CLTR	18	870	23.33	1	9	12.18	EARP
SH3KBP1	Q96B97	CLTR	15	665	66.77	2	15	46.32	ASP
EPS15	P42566	CLTR	13	896	49.00	4	14	23.10	ASP
NUMB	P49757	CLTR	12	651	61.29	5	13	37.48	ASP
CLTA	P09496	CLTR	10	248	60.08	3	3	44.76	COAT
DAB2	P98082	CLTR	10	770	74.16	6	24	49.74	ASP
CLTC	Q00610	CLTR	10	1675	1.07	0	0	0.00	COAT
SH3GL2	Q99962	CLTR	10	352	33.24	1	8	19.60	UCP
SYNJ1	O43426	CLTR	9	1573	33.76	3	12	24.92	EARP
ITSN1	Q15811	CLTR	9	1721	28.41	5	14	9.59	ASP
HGS	O14964	CLTR	8	777	55.86	4	12	31.15	ASP
AP2A1	O95782	CLTR	7	977	14.74	2	4	8.60	ASP
COPB2	P35606	COPI	6	906	9.16	1	3	5.08	COAT
AP2B1	P63010	CLTR	6	937	4.16	0	1	1.28	ASP
AP2M1	Q96CW1	CLTR	5	435	4.83	0	1	1.38	ASP
DNM3	Q9UQ16	CLTR	5	869	22.32	1	6	13.81	EARP

Same abbreviations used as in case of Table 1. CLTR stands for the clathrin route. Num. stands for number in the headers. Proteins are ordered according to the number of their off-pathway interaction partners.

All these results suggest that apart from some subunits of large complexes of coats and adaptors, most of the proteins showing moonlighting capabilities are often highly disordered and have several LDRs and many predicted DBRs. Among the three main routes, the clathrin pathway is the only one rich in such proteins, thus the vast majority of non-vesicle trafficking related interactions are mediated by this pathway. Further attesting to the functional adaptability of the clathrin route is that we observed the partners of proteins in this route being the most diverse with respect to their main pathways/molecular functions. For example, for the COPII proteins the 50 off-pathway interactions involve only 40 unique non-vesicle trafficking interaction partners (more COPII proteins can interact with the same off-pathway partner) out of which 15 are subunits of the nuclear pore complex alone (this is not surprising, since the ER membrane is continuous with the nuclear membrane, which allows for the free “flow” of membrane-anchored protein complexes between them) and there are also several lysosome-associated proteins and secreted ones.

### Structural disorder and binding regions of tissue-specific exons in vesicle trafficking proteins

All reported examples of TSEs for all budding- and fission-associated proteins in our data set were collected from Wang et al. [65]. The resulting 44 coding exons mapped onto 1550 residues in Ensembl transcripts. Structural disorder and disordered binding regions for the corresponding whole protein sequences were calculated (Table S4 in the Supplementary material).

Our results show that clathrin route associated proteins seem to be the ones which have most frequently acquired tissue-specific exons during their evolution: 33 of the 44 identified exons (75%) are located in 22 unique clathrin route associated proteins (∼31% of its proteins showing at least 1 TSE), while only 7 such exons were found for 5 COPII related proteins, 3 for 2 COPI associated proteins, and 1 in TMED2, a transmembrane cargo receptor shared between the COPI and COPII routes (25.8% of the COPII, and 13.6% of the COPI route proteins having at least one TSE). TSEs showing the strongest tissue specificity (measured by exon switch scores reported in Methods) are even more enriched in clathrin associated proteins, since out of the 23 TSEs with an exon switch score ≥ 0.5, 20 occur in clathrin proteins (∼87%) and only 3 in COPII proteins (one of these being shared with the COPI route).

According to IUPred predictions, 45% of the 1550 residues encoded by the TSE exons are located in disordered regions (the predictions were performed on whole proteins, and only TSE regions were taken into account from the predictions), almost twice the value for the complete human proteome (22.81%). According to ANCHOR predictions, there are 39 DBRs that are either completely encoded or overlapping with the protein regions corresponding to the 44 exons, and 4 additional binding regions were found to overlap with the 5 residue neighborhood of TSE boundaries, which are most probably also affected by the presence or absence of the given TSE.

The 33 TSEs present in the clathrin pathway proteins encode for a total of 970 residues. The disorder content of these TSE-encoded protein regions is higher in the clathrin route proteins (47%) than in the proteins associated with the other two routes (39.3%). 29 DBRs were found within the corresponding clathrin route related protein regions and the 4 binding regions that reside within 5 residues from the TSE boundaries are also found in association with these regions. The low number of TSEs in the proteins of the COPI and COPII routes do not allow statistical comparisons between the three pathways, but point to the fact that clathrin route proteins are far more prone to acquire such regions. Table 4 presents cases of strongly tissue specific TSEs (exon switch score ≥ 0.35) with at least 7 amino acids contribution to the protein chain.

**Table 4:** Structural disorder and binding motifs in the tissue specific exon encoded protein regions of the three main pathways.

Gene Name	ENSEMBL Transcript	Exon Switch Score	Route	Class	TSE location in protein	% disordered residues in TSE	More disordered than proteome reference (22.81%)?	DBR locations
AP1B1	ENST00000405198	1	CLTR	ASP	667–674	75%	Yes	N/A (649–666)
BIN1	ENST00000259238	1	CLTR	ASP	255–269	100%	Yes	246–270
PICALM	ENST00000341783	0.92	CLTR	ASP	420–469	18%	No	N/A
CLTB	ENST00000310418	0.9	CLTR	COAT	156–174	15.79%	No	155–177
AP1S2	ENST00000340245	0.8	CLTR	ASP	140–153	0%	No	N/A
CLTA	ENST00000242285	0.77	CLTR	COAT	180–192	0%	No	168–187
ARRB1	ENST00000420843	0.75	CLTR	ASP	333–341	0%	No	N/A (342–352)
TMED2	ENST00000438031	0.74	COPI/II	UCP	125–132	100%	Yes	N/A
BIN1	ENST00000351659	0.74	CLTR	ASP	174–204	0%	No	N/A
EPN1	ENST00000270460	0.73	CLTR	ASP	202–226	100%	Yes	204–223
BIN1	ENST00000393041	0.69	CLTR	ASP	305–340	100%	Yes	306–385
ARRB2	ENST00000381488	0.64	CLTR	ASP	8–18	0%	No	N/A
GGA1	ENST00000343632	0.62	CLTR	ASP	278–313	44.44%	Yes	279–299 (314–337)
ARRB2	ENST00000269260	0.59	CLTR	ASP	8–18	0%	No	N/A
SH3KBP1	ENST00000379716	0.57	CLTR	ASP	261–303	100%	Yes	256–271, 280–357
BIN1	ENST00000409400	0.57	CLTR	ASP	304–333	100%	Yes	273–350
SAR1A	ENST00000373239	0.54	COPII	EARP	20–59	0%	No	N/A
GGA1	ENST00000343632	0.5	CLTR	ASP	278–313	44.44%	Yes	279–299
EPS15L1	ENST00000248070	0.5	CLTR	ASP	794–862	94.20%	Yes	797–845, 853–864
SAR1A	ENST00000373239	0.49	COPII	EARP	1–59	0%	No	N/A
AX747833 (NUMB homolog)	ENST00000359560	0.46	CLTR	ASP	355–402	83.33%	Yes	358–380, 391–422
EPN1	ENST00000411543	0.46	CLTR	ASP	1–16	0%	No	N/A
ARFGAP3	ENST00000263245	0.42	COPI	EARP	355–399	33.33%	Yes	350–358
EPN3	ENST00000268933	0.37	CLTR	ASP	228–254	92.59%	Yes	236–250
DAB2	ENST00000320816	0.36	CLTR	ASP	230–446	99.08%	Yes	232–243, 253–267, 270–290, 309–316, 330–355, 358–378, 384–434, 446–464
DNM1	ENST00000341179	0.36	CLTR	EARP	875–881	100%	Yes	867–881

Same abbreviations used as in case of Table 1. CLTR stands for the clathrin route. Proteins are ordered according to the exon switch score defined for their TSE in Wang et al [65]. DBR locations not overlapping with the TSE encoded regions themselves, but starting within 5 residues from the boundaries of those are listed in brackets.

### Domains typically surrounded by disordered regions

We identified at least one Pfam ‘entity’ (143 families; 153 domains; 3 motifs; 9 repeats) for 238 vesicle trafficking proteins in human. There were only 10 proteins for which no domain or family could be assigned. We further analyzed the Pfam patterns of highly disordered proteins, namely those with at least 70% disorder content or a high ratio of LDR residues (≥mean plus 2 times standard deviation or ≥50%). In some cases, such proteins are assigned to Pfam families based on evolutionary conservation, and yet they do not contain any folded domains. Such is the case of all the complexins (1–4), which belong to the synaphin family and are highly disordered (disorder content 76–98%) according to our predictions. Another example is the family of clathrin light chains, where both proteins show very high predicted disorder content (60% and 74%) and have no folded domains assigned.

Our data *clearly* show that there are several folded domains that are typically located in highly disordered proteins. These structured “islands” are usually surrounded by extended disordered regions on either or both of their sides, and tend to be the sole domain of the protein. Examples of such kind of domains include the ENTH (epsin N-terminal homology) domain, the PID (phosphotyrosine interaction domain), the Sec16 domain, and the muHD (muniscin C-terminal mu homology domain), among others.

The highly disordered epsin type clathrin adaptors (1–3) have the unique ENTH domain at their N-terminus, which serves as a membrane interacting module, while the remaining part of the protein is completely disordered. Epsins 1 and 3 also contain UIM motifs (Ubiquitin Interaction Motifs) within their disordered regions along with the many identified adaptor protein- and clathrin-binding motifs [21], [35], [38]. Another clathrin adaptor, DAB2, has also one single domain at its N-terminus, the PID, which roughly coincides with the only structured region of this highly disordered protein (74% disorder content). NUMB (61.21%) and DAB1 (51.36%) also share this PID domain comprising the only structured region of these proteins. The muHD domain is also coupled with a long disordered segment, in this case from the N-terminal side. This domain is present in three disordered adaptors from the clathrin system: SGIP1 (SH3-containing GRB2-like protein 3-interacting protein 1, disorder content 62.68%), FCHO1 (FCH domain only protein 1, d.c. 47.58%) and FCHO2 (FCH domain only protein 2, d.c. 34.57%). Another example of these structured island domains is the Sec16 domain found in Sec16A and Sec16B (Protein transport protein Sec16A and B). Located approximately in the middle of these huge proteins (∼2000 residues), it is surrounded by highly disordered termini on both sides (the structural characteristics of Sec16A will be further discussed in the next section). The ArfGAP domain is also usually located on the N-terminal end of the long, considerably disordered ArfGAP proteins (d.c. 36.63–52.22%).

Apart from the previously mentioned domains, we have found others, which are often surrounded by variable long disordered regions, but are also present in proteins that tend to have less disorder content. The BAR (Bin-Amphiphysin-Rvs) domain – involved in membrane curvature sensing – is present in the highly disordered amphiphysin (d.c. 60.58%), but also in endophilins (A1-3, B1-2), which have substantially variable disorder content (d.c. 7.12–35.60%). The protein kinase domain is present in AAK1 (AP2-associated protein kinase 1; d.c. 58%), but it is often part of other less disordered kinases as well.

In summary, the vast majority of the domains that are always surrounded by highly disordered regions belong to clathrin pathway associated adaptor and sorting proteins (all the epsins, NUMB, DAB1, DAB2, SGIP1, FCHO1 and FCHO2). Their structural properties – having a single folded domain located at one of their termini, while the rest of their chain is highly disordered with embedded functional motifs – make them excellent candidates for the fly-casting mechanism. Additionally, previous studies have shown that these adaptors are able to form extended adaptor networks on the surface of the budding vesicle, some of them being responsible for recruiting clathrin as well [20], [21], [35]. According to the ANCHOR prediction method, and as it was pointed out in previous works [21], [38], these long disordered regions contain a plethora of different binding motifs, which can facilitate specific interactions between the adaptor proteins or with clathrin, but also with other components of the system or possible “off-pathway” interaction partners.

**Figure 4:**
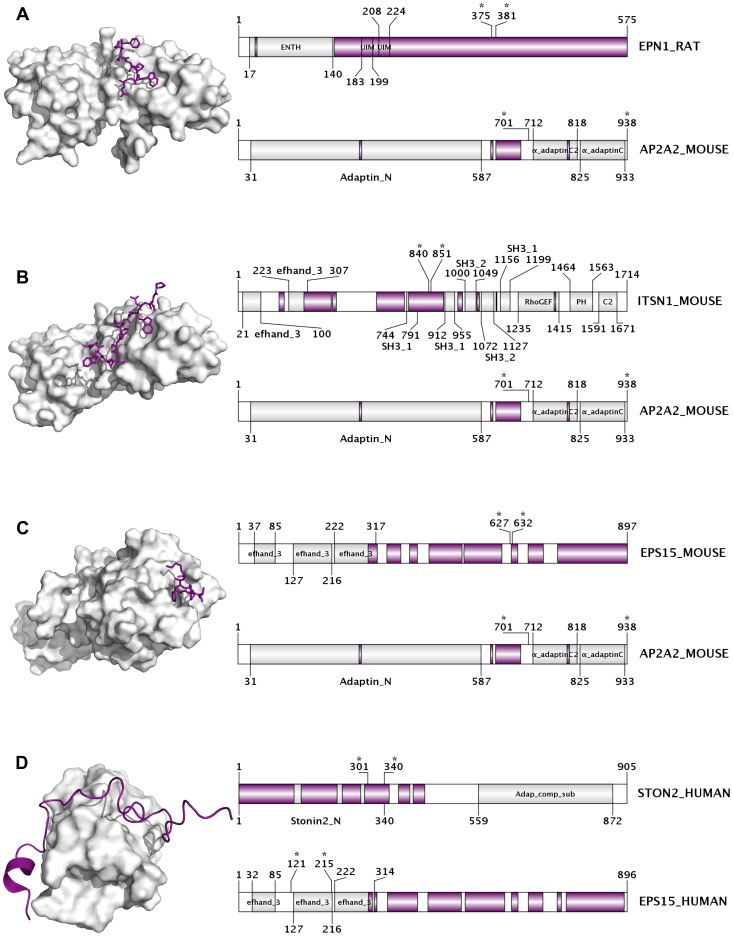
Interactions between pairs of clathrin-associated adaptor proteins. Complexes formed between clathrin-associated adaptor proteins are presented, in which one partner interacts with a region predicted to be structurally disordered in the unbound form. On the first three panels, the α2 subunit of mouse Ap-2 is the folded partner interacting with (A) rat epsin-1 (PDB 1KY6), (B) mouse intersectin-1 (PDB 3HS8); and (C) mouse EPS15 (Epidermal growth factor receptor substrate 15, PDB: 1KYF). In panel D, a relatively long disordered segment of human stonin-2 interacts with one folded EF-hand domain of human EPS15 (PDB: 2JXC). In each panel, the structure of the complex is depicted on the left and a domain map for each partner is depicted on the right. The top domain map represents the partner that is binding through the structurally disordered region. In panels A to C, the disordered peptides are represented with sticks (purple) while the folded partner is shown in surface representation (white). In panel D, the long disordered segment of human stonin-2 is shown in cartoon representation. For each protein, the domain maps indicate the names and locations of the known Pfam domains (predicted by the PfamScan method), and are shown in gray segments. Regions predicted to be disordered by IUPred are marked in purple segments; regions present in the PDB structure are marked by stars.

To further investigate the role of disordered binding regions in building the adaptor network, we performed a systematic PDB search looking for clathrin coat specific protein complexes. We found several structures where the interaction of two clathrin adaptors is shown, and one of the partners uses its disordered binding regions to bind to the folded domain of the other one. In case of the multisubunit AP-2, one of the most studied key players of endocytosis, two long disordered regions connect the two α-adaptin ear domains to the major part of the huge complex. The recognition of cargo sorting signals is done by the major part, while the principal clathrin-binding region is located in the disordered β2-adaptin hinge [21], [38]. The two α-adaptin ear domains are favoured targets of disordered tails of other clathrin adaptors and accessory proteins [70]. We found several distinct complexes showing these interactions (Figure 4 A, B and C). In these complexes, usually very short peptide constructs (lengths ranging between 6–12 residues) of disordered regions in the partner proteins bind to the α-adaptin ear domain. In addition, we found an interesting case where a relatively long, highly disordered region of human stonin 2 binds to one of the small folded EF-hand domains of human EPS15 (Epidermal growth factor receptor substrate 15) (Figure 4 D).

We also found structures where other non-adaptor clathrin pathway associated proteins interact with AP-2 or clathrin via their disordered segments. Amphihpysin, for instance, interacts with both (PDB IDs 2VJ0 and 1UTC) via two different disordered binding regions located in the long disordered segment following the BAR domain. Proteins from the EARP functional group also bind AP-2, in case of synaptojanin 1, two distinct constructs were shown to bind the α-adaptin domain (PDB ID: 1W80), while PIP5K1C (Phosphatidylinositol 4-phosphate 5-kinase type-1 gamma) interacts with the β subunit (PDB ID: 3H1Z).

### Identification of orthologous protein pairs and analysis of their disorder content

We identified 56 human proteins that could be successfully matched to a yeast protein from our dataset using the Inparanoid 7.0 method. We focused on orthologous pairs involved in the budding and fission associated functional groups because they show higher abundance of disordered regions, they were the ones that could be reliably grouped according to the main routes and hence better distinguishing between those. We filtered these pairs for at least one of their members showing considerably high disorder content (>30%). From the resulting 8 protein pairs (this relatively small number well indicates the lower evolutionary conservation levels observed for disordered protein regions in general) one showed very similar disorder content (less than 5% difference); 5 pairs showed more disorder in the human ortholog than in the yeast ortholog; and in two cases the yeast protein showed higher disorder content.

**Figure 5:**
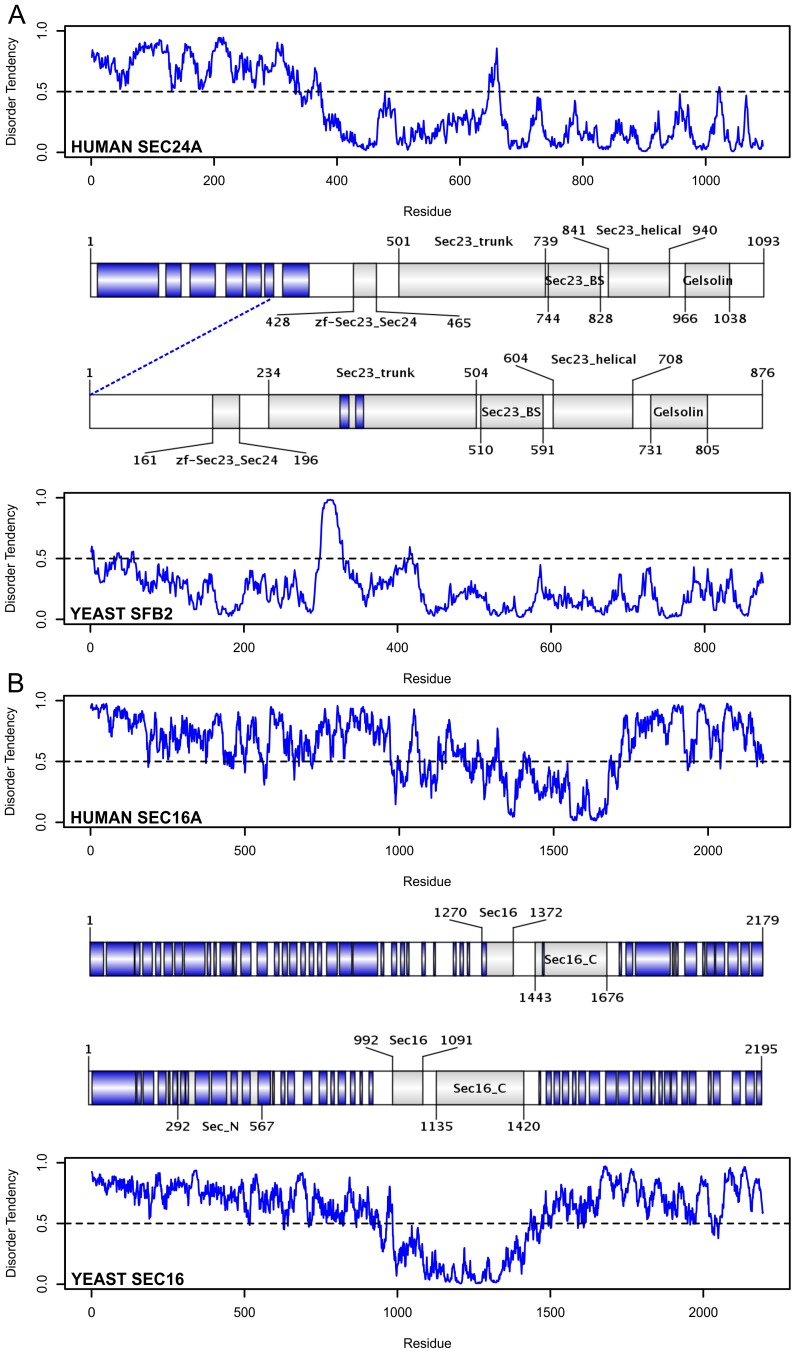
Structural comparison of orthologous proteins involved in vesicle trafficking. The structural characteristics of two orthologous protein pairs from the COPII vesicle trafficking system are presented. On panel A, the moderately disordered (34.13%) human Sec24A COPII adaptor subunit is compared to its virtually ordered (5.94%) yeast ortholog (SFB2, Sec24 related protein). On panel B, the highly disordered human Sec16A (71.41%) and yeast Sec16 (74.44%) proteins are presented. The disorder pattern predicted by the IUPred method is plotted for the members of each pair (blue curve), where the black dashed line (y = 0.5) represents the cut-off between order and disorder. Residues with disorder tendency above this cut-off are considered to be disordered. A domain map is also presented for each protein. In it, the location and names of their identified Pfam domains (gray segments) and their disordered binding regions predicted by the Anchor method (blue segments) are shown. In each panel, the human ortholog is depicted in the top part: the disorder prediction curve followed by its corresponding domain map. The bottom part of each panel is a specular representation of the corresponding yeast ortholog: the disorder curve is topped by the domain map. The corresponding disorder curves and domain maps are fitted in length so that structural information can be directly reflected on the disorder pattern. The blue dashed line connecting the domain maps in panel A shows the position in the human ortholog which corresponds to the N-terminal end of its yeast ortholog.

We analyzed two protein pairs in detail (Figure 5). The first pair shows the largest difference in disorder content among all pairs: human Sec24A (protein transport protein Sec24A) and yeast SFB2 (SED5-binding protein 2) with a sequence identity 22.66% (Figure 5A). Here, the human sequence is considerably longer due to a long, disordered segment at the N-terminal region, which is missing in the yeast ortholog. Being also abundant in predicted disordered binding regions (shown in blue), this region might be a result of adaptive evolution. In fact, this subunit of the COPII coat-adaptor complex is important for the recognition and binding of the cargo (transmembrane cargo proteins, and transmembrane cargo receptors of soluble proteins) [32]. Given that the repertoire of possible cargo proteins transported from the ER to the Golgi is considerably higher in human than in yeast, the presence of additional binding regions in the human ortholog could make sense.

In the second pair, both proteins are highly disordered: human Sec16A (Protein transport protein Sec16A) and yeast Sec16 (COPII coat assembly protein SEC16), and according to their predicted disorder patterns (Figure 5B), despite the rather low overall sequence identity (14.07%), their disordered nature is well conserved. Both members are extremely long (>2000 residues), highly disordered proteins (74.44% and 71.4% disorder content in the yeast and human orthologs, respectively). Their domain maps show that the huge disordered regions surrounding the Sec16 (and Sec16_C) domains are almost entirely covered by DBRs. These two proteins are highly similar in length, and can be considered well conserved from the structural point of view. Their preserved disordered nature, with plenty of DBRs (54.8%) and an even higher ratio of residues located in LDRs (62.1%) is in a good agreement with their essential roles in COPII vesicle assembly [69] and cargo selection. For the coat assembly, the long disordered regions can be especially advantageous, because – being able to bridge very long distances through the fly-casting mechanism – they can reach for the components of the vesicle coat from the surrounding environment, and help them to acquire proper orientation for the assembly. In case of the clathrin system, it is usually the group of adaptor proteins that is responsible for this function. The same proteins can perfectly utilize their disordered regions to form the adaptor network on the vesicle surface and to attach the clathrin chains to the surface of this network as well. In the COPII system, however, the adaptors are part of the multisubunit adaptor-coat complex, and the two subunits playing the adaptor role are certainly not disordered enough to fulfil these roles. Hence, there is definitely a need for a large disordered protein, like Sec16 (and its homologs) to orchestrate the assembly of the coat components, especially when large distances need to be spanned.

## Discussion

Vesicle trafficking routes have fundamental roles in the eukaryotic cell, providing the possibility of targeted macromolecule transport between the various intracellular compartments and also the cell and its environment. The COPI, COPII and clathrin-mediated vesicle trafficking routes comprise the major part of the transport network, being responsible for the different types, locations and directions of traffic involved in endocytosis, the early and late secretory pathways and the retrograde Golgi-ER transport. Despite their similarities, there are fundamental functional and evolutionary differences that strongly distinguish these routes, yet the structural characteristics that could account for these differences have not been previously described.

In this work, we provided a systematic assessment of the potential functional involvement of structurally disordered protein regions in the main vesicle trafficking systems. Based on the functional requirements of their proteins and the inherent advantages that structural disorder could offer them, we expected such systems to heavily rely on disordered protein regions. Such regions have been widely recognized to be abundant in proteins related to signalling and regulatory roles [41], [42]. They can act as flexible linkers between structured domains to enhance their free movement and rotations [71] providing the possibility for large, multidomain proteins to acquire multiple supertertiary structures [72]. Due to their increased accessibility all types of proposed protein-protein interaction motifs [73] and posttranslational modification sites [47], [48] tend to reside in disordered regions, hence they are also frequently involved in molecular recognition and regulatory functions [21], [38]. Protein disorder also provides many advantages in fine-tuning the kinetics and thermodynamics of molecular recognition events [74]. Moreover, extended disordered regions are especially useful in the assembly of large macromolecular complexes [52], similar to the ones involved in vesicle trafficking.

The different measures of structural disorder that were used to describe the abundance and location of such protein regions in vesicle trafficking proteins allowed us to distinguish between major functional roles, in which disordered regions could be involved. While the overall disorder content of proteins provided a broad picture about the dependence of the given functional group or trafficking route on structural disorder, the ratio of residues located in predicted DBRs helped to estimate the involvement of these regions in protein-protein interactions. In those cases where the ratio of residues in LDRs is considerably more than those in DBRs, we could speculate that besides promoting protein-protein interactions, disordered regions might also serve as flexible linkers between structured domains, or as long spacers, assisting in fly-casting mechanism by providing the possibility for the motif-rich parts to reach farther.

Despite the heterogeneity of proteins in the three major vesicle trafficking routes, we found that the proteins of these systems followed similar overall tendencies of structural disorder in the two species. The overall statistic comparison of human and yeast proteins showed that proteins involved in vesicle trafficking are only slightly more disordered in human. The equivalent functional groups in the two species showed very similar disorder contents, which reflect the well-conserved nature of vesicle trafficking proteins. These results are consistent with previous works showing that intrinsic disorder is not necessarily correlated with organism complexity [75], [76]. In this case, structural disorder is intrinsic to the biological process rather than depending on the complexity of organisms, which also highlights the role of disorder in proteins involved in vesicle trafficking.

The importance of disordered regions in vesicle trafficking is also well reflected by the fact that almost all the main functional groups have highly disordered (>50%) members in both species. The big differences observed between the disorder contents of different groups nonetheless imply that certain functions require the presence of disordered protein segments more than others.

Not surprisingly, most coat proteins are mainly structured, since they tend to fold into rigid cage-like structures on the surface of all types of vesicles. Despite much different cage architectures, they all contain the same two types of folded building blocks: α-solenoids and β-propellers. Only the group of clathrin light chains (CLCs) was predicted to be largely disordered, while the different Sec31 COPII coat subunits possess a long disordered segment between their structured domains. The dynamic nature of the highly disordered CLC has an important role in the regulation of clathrin lattice assembly through allowing for large conformational switches, which also influence the conformation of the heavy chain knee regions [11]. The coat assembly is also influenced by the various interaction partners of the light chain, like HIP1 [77], [78]. In our view, the flexibility of the CLC chain could be essential for promoting the right packing process of the extraordinarily tight, highly overlapping, clathrin triskelion cage [5], [6], which far exceeds the packing density of the other two types of coat complexes [6], [10]. Furthermore, the CLCs could be important in the ability of self assembly as well [5]. Sec31 COPII coat subunits all have a very long predicted disordered region matching the proline-rich unstructured segment described in the literature as a flexible linker between the two long α-solenoid repeat regions [7] that mediates the interaction with the Sec23/24 subcomplex [69].

Some of the proteins involved in fusion-related functions also showed a considerable amount of disorder, although most of the functional groups (like the MSTC, OFRP and NTSR groups) here had rather low disorder content. The SNARE group was the most disordered among these in both species, since the different SNARE homology domains are unfolded in their monomeric form [22], [23], which was well-detected by the applied disorder prediction method. As it was pointed out in Figure 2, many of the SNARE proteins, namely the syntaxin family members, also have disordered N-terminal regulatory segments that allow their regulatory binding partners (SM proteins) to modify their functions [23].

The NTSR group, although showing relatively low overall disorder content, contains the most disordered protein family of our data set, that of complexins. These SNARE regulatory proteins are predicted to be almost completely unfolded. In their complexes the ‘central helix’ of complexins is interacting with one SNARE complex, while their ‘accessory helix’ forms a bridge to another SNARE complex, occupying the empty v-SNARE binding site to inhibit vesicle fusion. Their accessory helix was thought to compete with the v-SNARE homology domain for binding the prefusion t-SNARE complex, but recently it was shown rather to help organizing the t-SNAREs into a zigzag topology that is incompatible with fusion (PDB: 1KIL, 3RLO) [79], [80]. Considering their function, complexins prevent SNAREs from neurotransmitter release until an action potential arrives at the synapse. They are essential grappling/clamping proteins [81] that help stabilize SNAREs in an active, but yet frozen state, and only release them when synaptotagmins give a Ca^2+^-induced signal for this [23], [82]. The mechanism by which synaptotagmins can pass the information about the Ca^2+^ signal to complexins is not yet fully understood. According to our predictions, complexin regions forming the helixes in the complexes are unfolded in their monomeric state, just like the SNARE coiled coil homology domains that they are mimicking.

The group of “adaptor and sorting proteins” showed the highest number of extremely disordered members, especially because of the non-complex-forming clathrin adaptors. Although it contains many fully structured complex subunits as well (especially due to the many, highly similar subunits of the four different AP complexes in the clathrin route), it is quite evident that intrinsic disorder has a fundamental role in maintaining many of the functions carried out by this group, such as linking the coat scaffold to the cargo and to the membrane, helping vesicle coat assembly by binding the coat subunits, and communicating with other accessory proteins. The dependency on structurally disordered regions must be the largest in case of the clathrin system, since most of the individual clathrin adaptors, many of the accessory proteins and also some of its enzymes (like synaptojanins) have extremely long disordered tails with a large collection of several protein interaction motifs. Also, when searching for those solitary folded domains, which behave like structured “islands”, surrounded by extended disordered regions on either or both sides, most of the examples that we could identify belonged to the ASP group of the clathrin-mediated system. These proteins seemed to be the best candidates for the fly-casting mechanism, since they could behave as a fishing stick, their folded domain being fixed to the surface of the vesicle or to bigger protein complexes, while their disordered, flexible tail can freely reach for their various partner proteins over relatively long distances. This binding mode can be especially advantageous in the vesicle assembly process because it may enhance the speed of recognition and bring the coat components into close proximity to the surface of the budding vesicle. Unstructured proteins have larger capture radius that helps them efficiently utilize their many interaction motifs. Additionally, we collected several examples from the PDB that provide structural evidence for protein interactions mediated by the induced folding of disordered binding regions in clathrin system related proteins. Many of these structures showed the same domain, the AP-2 α-adaptin ear domain, facilitating specific interactions with disordered binding motifs of its partner proteins (Figure 4). The partners were not exclusively adaptor proteins in this case; there are also structures about synaptojanin-1 and amphiphysin interacting with the ear domain. We found other examples of clathrin system related complexes as well, like human stonin 2 binding to the EPS15 EF-hand domain. All these observations are in a very good agreement with the previously described extended, dynamic protein network on the surface of clathrin coated pits [20]. The composition of this network is probably highly variable in a localization-, route-, and maybe cargo-specific manner, with several different functional groups represented among its members.

The functional importance of disordered regions was also well reflected by the conserved nature of their location, while the variability in their length and their low sequence similarity showed their increased adaptability and tolerance against mutations compared to folded domains. When investigating orthologous protein pairs from human and yeast, the location of disordered regions was found to be quite conserved, while their length appeared to be more subject to evolutionary change. In case of the Sec16 pair, the long disordered regions surrounding the structured domains were very well preserved, and even the lengths of the two proteins were highly similar. Since almost the entire length of the two long disordered “arms” of the proteins is covered by predicted disordered binding sites in a well-conserved way, their essential role in the initiation of the COPII coat assembly is very likely. The level of conservation in these regions seems to heavily depend on the specific functional needs of the given protein. In case of the Sec24 orthologous pair, for instance, the human sequence has a considerably long N-terminal unstructured region, which is almost completely missing from the yeast counterpart. The presence of numerous predicted disordered binding regions implies that this region is the result of adaptive evolution. Since this protein is a key player in cargo recognition and binding, which obviously involves a larger repertoire of possible cargos in human, the emergence of such adaptive regions could provide indisputable benefits.

Our results showing that the clathrin system is significantly more disordered than the COPI and COPII systems not only imply the larger dependence of this system on disordered protein segments and support the concept of highly dynamic networks formed by its proteins, but also explains much about the differences between the three routes from the evolutionary point of view [35]. Disordered regions not only have conformational freedom but also a kind of evolutionary freedom. Their increased tolerance against mutations gives them the possibility of fast evolutionary changes, providing exceptional adaptability. As already mentioned, the clathrin-mediated system shows marked plasticity and robustness compared to the other two systems. There are many observations emphasizing the increased adaptability of this system as well. It shows many species-specific characteristics [27], [31], and it has been extensively modified to assist other specialized pathways. Adaptors and the clathrin itself, for instance, are often manipulated to create novel types of organelles, such as the rhoptry secretory organelle in *Toxoplasma gondii* [83], the contractile vacuoles of Dictyostelium species [84], special vesicles for odorant receptors transport of *Caenorhabditis elegans* [85], and the machinery for sorting proteins to the basolateral plasma membrane of vertebrate epithelial cells [86], among others. Biogenesis of synaptic vesicles in animals and human is also performed by endocytic adaptors [87]. Similarly, there are other organelles as well that require these adaptors for their maturation [88]. Apart from the species and tissue-specific inventions, clathrin system adaptors are also frequently used for various functions during embryonic development [88], [89] and often responsible for mitotic moonlighting functions of many kinds [90].

Taken together, these observations on the many different adaptive changes manifest on clathrin-route related proteins strongly support the idea that it has been favoured by evolution over the two other main trafficking systems. Until now, the structural background of this phenomenon had not been established. In this study we show how structural disorder found in this system underscores its exceptional adaptability. We analyzed the moonlighting abilities and tissue specific functions of the proteins in the three main vesicle trafficking routes, as they are both strong indicative measures of adaptability. The clathrin route clearly stands out, having the highest number of non-vesicle trafficking interaction partners and the most verified tissue specific exons. The correlation between these abilities and the disordered nature of the corresponding proteins/protein regions in general had been previously suggested [49], [51], and here we demonstrate this positive correlation specifically for vesicle trafficking proteins.

Clathrin-route associated proteins have significantly more off-pathway interactions than COPI- and COPII-route associated proteins. Out of the most “interactive” 21 proteins, 19 are clathrin-route related, many of them having disorder regions (at least 30 consecutive residues and/or higher disorder content than the whole proteome reference value). More than one third of all off-pathway interactions of the three routes are mediated by only 9 non-complex-forming clathrin adaptors, most of which have LDRs densely covered by predicted DBRs and/or are highly disordered, meaning that more than half of their residues reside in disordered regions. The clathrin-route proteins also show more ability to maintain tissue specific functions than the proteins in the other two routes. 75% of all TSEs were found in clathrin-route associated genes, together with approximately the same ratio of TSE encoded predicted DBRs residing in clathrin proteins. TSE-encoded protein regions are almost twice as disordered as the proteome average, and they have an increased capacity to host interaction sites (one in every 40 residues in our dataset). These findings are in good agreement with the general view that TSE-encoded protein regions are enriched in protein disorder [49] and differentially spliced exons in general, and tissue specific ones in particular, are prone to specifically rewire interaction networks by coding for protein regions enriched in short linear motifs/interaction sites [49], [50], [91]. Our results also show that TSE-encoded regions in the clathrin-route associated proteins are more disordered than the rest (encoded by the other two routes), which further points to the enhanced dependency of this route on structural disorder and the related advantages disorder offers.

In summary, we found many functional modalities enabled by disordered regions to be present in vesicle trafficking proteins. These include regulatory roles, the use of flexible linkers, mediating protein-protein interactions with proteins of the same route or others, or the quick assembly of large macromolecular complexes by fly-casting. Taken together, our results provide compelling evidence for the functional involvement of structural disorder in the main vesicle trafficking systems. The presence of highly disordered proteins in almost all the main functional groups of vesicle trafficking proteins emphasized the unquestionable importance of disorder for this cellular process in general. The remarkable differences in its abundance between the three main trafficking routes, however, provided structural background for long standing observations on the functional and evolutionary differences of these systems.

## Supporting Information

Table S1 **Vescile trafficking-related human proteins.** All human proteins related to vesicle trafficking, with various measures of predicted protein disorder.
(XLS)

Table S2 **Vescile trafficking-related yeast proteins.** All yeast proteins related to vesicle trafficking, with various measures of predicted protein disorder.
(XLS)

Table S3 **Off-pathway interaction partners of vesicle trafficking proteins.** The number of high confidence, non-vesicle trafficking related (off-pathway) interaction partners of proteins of the three vesicle-associated pathways in humans.
(XLS)

Table S4 **Tissue-specific exons (TSEs) in budding- and fission-associated human proteins have been collected from Wang et al.** [**65**] **.** The resulting 44 coding exons are mapped onto Ensembl transcripts, and their various disorder parameters are given.
(XLS)
